# Hi-C analysis of genomic contacts revealed karyotype abnormalities in chicken HD3 cell line

**DOI:** 10.1186/s12864-023-09158-y

**Published:** 2023-02-07

**Authors:** A. Maslova, V. Plotnikov, M. Nuriddinov, M. Gridina, V. Fishman, A. Krasikova

**Affiliations:** 1 Saint Petersburg State University, Saint Petersburg, Russia; 2 Institute of Cytology and Genetics SB RAS, Novosibirsk, Russia

**Keywords:** HD3, A/B compartments, Avian karyotype, Chicken cells, Chromosome rearrangement, Chromosome translocation, Avian erythroid differentiation, Genome architecture, Hi-C, Topologically associating domains

## Abstract

**Background:**

Karyotype abnormalities are frequent in immortalized continuous cell lines either transformed or derived from primary tumors. Chromosomal rearrangements can cause dramatic changes in gene expression and affect cellular phenotype and behavior during in vitro culture. Structural variations of chromosomes in many continuous mammalian cell lines are well documented, but chromosome aberrations in cell lines from other vertebrate models often remain understudied. The chicken LSCC-HD3 cell line (HD3), generated from erythroid precursors, was used as an avian model for erythroid differentiation and lineage-specific gene expression. However, karyotype abnormalities in the HD3 cell line were not assessed. In the present study, we applied high-throughput chromosome conformation capture to analyze 3D genome organization and to detect chromosome rearrangements in the HD3 cell line.

**Results:**

We obtained Hi-C maps of genomic interactions for the HD3 cell line and compared A/B compartments and topologically associating domains between HD3 and several other cell types. By analysis of contact patterns in the Hi-C maps of HD3 cells, we identified more than 25 interchromosomal translocations of regions ≥ 200 kb on both micro- and macrochromosomes. We classified most of the observed translocations as unbalanced, leading to the formation of heteromorphic chromosomes. In many cases of microchromosome rearrangements, an entire microchromosome together with other macro- and microchromosomes participated in the emergence of a derivative chromosome, resembling “chromosomal fusions'' between acrocentric microchromosomes. Intrachromosomal inversions, deletions and duplications were also detected in HD3 cells. Several of the identified simple and complex chromosomal rearrangements, such as between GGA2 and GGA1qter; GGA5, GGA4p and GGA7p; GGA4q, GGA6 and GGA19; and duplication of the sex chromosome GGAW, were confirmed by FISH.

**Conclusions:**

In the erythroid progenitor HD3 cell line, in contrast to mature and immature erythrocytes, the genome is organized into distinct topologically associating domains. The HD3 cell line has a severely rearranged karyotype with most of the chromosomes engaged in translocations and can be used in studies of genome structure–function relationships. Hi-C proved to be a reliable tool for simultaneous assessment of the spatial genome organization and chromosomal aberrations in karyotypes of birds with a large number of microchromosomes.

**Supplementary Information:**

The online version contains supplementary material available at 10.1186/s12864-023-09158-y.

## Background

Cells of the same type within a given population or tissue often display similar gene expression programs to one another and this, in turn, is related to specific profiles of epigenetic modifications and spatial genome organization. Gene activity is tightly linked to three-dimensional (3D) chromatin folding that has been revealed in multiple cell types and at different genomic length scales by chromosome conformation capture methods (C-methods), including Hi-C as a full genomic variant [1, 2]. Average nuclear chromatin interactions, as depicted in Hi-C heatmaps, are more frequent within local self-interacting topologically associating domains (TADs), structured via dynamic looping of chromatin regions, and within higher-scale active (A) and inactive (B) compartments, formed by phase separation of chromatin with distinct molecular signatures [3, 4]. Significant changes in TADs and compartments, although to different extents, were demonstrated in different cell types, both primary and cancer, and under different conditions through differentiation, development and malignant transformation [5–8]. Disruption of TAD borders associated with cancers and other diseases may cause deregulation of genes and drastic phenotypic effects [9–15].

Capturing the spatial organization of the genome is also widely employed to identify genome structural variations (SVs) in primary cancers and cancer cell lines [16–22]. SVs are generally deduced from anomalous patterns of inter- or intrachromosomal contact frequencies compared to the normal genome. In this way, different chromosomal aberrations, such as translocations (balanced or unbalanced), deletions, inversions and duplications, can be identified. Nevertheless, while most SVs can be found by visual inspection of Hi-C heatmaps, many computational algorithms have also been developed, with varying abilities to detect SVs [18, 23–25]. A distinct advantage of Hi-C over whole-genome sequencing is simultaneous recognition of both genome topological information and de novo detection of large-scale SVs using even low-depth Hi-C maps [19, 26].

Continuous cell lines with unlimited lifespan provide a useful source in biomedical research for in vitro investigation of cell phenotypes in different experimental conditions, including those simulating the in vivo state during viral invasion, oncogenic transformation and cell differentiation [27, 28]. The chicken LSCC-HD3 cell line (hereafter referred to as HD3) was generated from erythroid precursors transformed by a temperature-sensitive mutant of avian erythroblastosis virus (*ts*34AEV) [29]. The HD3 cell line preserved its nondifferentiated state and proliferative potential in culture under “permissive” temperature (36 °C) and was able to start differentiation under a temperature shift towards 42 °C, when the virus oncogenes were inactivated [29]. HD3 was used to study the molecular basis of oncogenic transformation by AEV [30, 31] and, importantly, served as a valuable model to analyze signaling pathways and gene regulation following erythroid differentiation in a chicken model [32–36]. Chromosome conformation capture variants (4C-seq and 5C) were previously applied in the HD3 cell line to analyze 3D genome organization in several chromosomal regions, specifically focusing on the α-globin gene cluster crucial for erythroid differentiation [37, 38]. Well-discerned TAD structures and A/B compartments form in the chicken α-globin locus and flanking regions [38]. Moreover, whole-genomic approaches demonstrated conservation of these topological features of the genome between birds and mammals [39, 40]. However, genome-wide profiles of TADs and A/B compartments for HD3 cells have not yet been available. General genome instability associated with AEV transformation and chromosome SVs in the HD3 cell line have not yet been characterized. It should be noted that the identification of chromosomal rearrangements in chicken cell lines by karyotyping is hampered by a large number of chromosomes (2n = 78), including numerous microchromosomes, which are small, indistinguishable by banding and frequently lost during metaphase spread preparations [41–43]. On the other hand, the number of commercially available probes is limited for chicken compared to mammalian species.

Here, we aimed to obtain a Hi-C chromatin contact map for the chicken HD3 cell line to characterize its genome organization, reveal chromosome rearrangements and verify them by FISH.

## Methods

### Cell culture

The HD3 cell line was grown in high-glucose (4.5 g/l) DMEM (Capricorn Scientific), containing stable glutamine and sodium pyruvate, supplemented with 8% fetal bovine serum (Gibco), 2% chicken serum (Gibco) and 50 µg/ml gentamicin at 37 °C and 5% CO2. Cells were passaged 1:5 every 3 days. For metaphase chromosome preparations, cells were grown in full DMEM with 0.1 µg/ml colcemid for 3 h and harvested by centrifugation. Cells were treated with 0.075 M KCl hypotonic solution for 20 min at 37 °C, collected by centrifugation and fixed in cold 3:1 methanol-acetic acid solution according to the standard procedure. Metaphase chromosome spreads were obtained by dropping a fixed cell suspension onto hot slides in a water bath as described earlier [44].

### Hi-C in situ protocol

HD3 cells were counted and resuspended in serum-free DMEM up to 1 × 10*6 cells/ml. Cells were fixed in 1% PFA for 10 min with constant mixing. PFA was quenched by 125 mM glycine for 10 min, and the cells were centrifuged at 800 g for 10 min at 4 °C and washed with DPBS. Cell pellets were snap-frozen in liquid nitrogen for further processing. Hi-C libraries were prepared from 5 × 10*6 fixed cells according to the Hi-C 2.0 protocol [45] with minor modifications using two replicates of cross-linked HD3 cells. Cells were lysed on ice in 10 mM Tris–HCl (pH = 8.0), 10 mM NaCl, 0.2% Igepal CA-630 and protease inhibitor cocktail. The cell pellets were washed twice in NEB 3.1 containing 0.1% SDS. Chromatin was digested with *Dpn*II (NEB) at 37 °C, and 5'-overhangs were filled with biotinylated dCTP. Chromatin ligation was conducted at 16 °C overnight. Crosslinks were reversed at 65 °C overnight in the presence of proteinase К (NEB). After DNA extraction, biotin-dCTP was removed from unligated (dangling) ends by incubation with T4 DNA polymerase at 20 °C for 30 min. DNA was sonicated (Covaris M220) into 200–400 bp fragments. After sonication, DNA fragments were further size-selected twice with AMPure beads. Biotin pulldown for library enrichment of ligation junctions was performed using Dynabeads MyOne Streptavidin C1 (Thermo Fisher Scientific). Finally, libraries were assembled using the KAPA Hyper Prep Kit (Roche) and KAPA Single-Indexed Adapter Kit Set A (Roche) in 9 PCR cycles. Hi-C DNA libraries were sequenced in paired-end read (PE75) mode on an Illumina HiSeq 2500 platform. Sequencing reads were filtered, mapped to the GalGal5 genome (Gallus_gallus-5.0, GCF_000002315.4) and assembled in the Hi-C heatmap using Juicer tools [46].

### Eigenvector decomposition and TAD calling

Eigenvector decomposition of Hi-C matrices was performed via Juicer tools. To compare compartment distribution between the cell types, we used 50 kb binned heatmaps to calculate the Pearson correlation coefficient. For domain calling, we applied the Armatus [47] algorithm to the HD3 Hi-C heatmap binned at 50 kb resolution and parameter γ = 0.2–0.3. Hi-C heatmaps, TAD annotations and compartment boundaries for chicken embryonic fibroblasts and granulosa cells from preovulatory follicles were obtained from Fishman et al., 2019 [39] and Li et al., 2022 [48], respectively. To compare sets of domains between different cell lines, we either directly compared the genomic coordinates of domain borders at an accuracy level of 50 kb, which corresponds to 1 bin, or used variation of information coefficient.

### Hi-C map analysis and detection of translocations

Normalized Hi-C maps were visualized and analyzed via Juicebox [49], including Juicebox assembly tools [50]. To identify the regions of interchromosomal breakpoints in the chromosome-to-chromosome view of the Hi-C heatmap, we binned the Hi-C map at 5 kb resolution. The detected translocation breakpoints were further verified by HiCtrans [23] with the following parameters: resolutions 1, covq 0.1, minzscore 1, relevel YES, precheck 1. For HiCtrans processing (https://github.com/ay-lab/HiCtrans), we used contact matrices at 50 kb resolution. Intrachromosomal rearrangements, including deletions, inversions and duplications, were detected through visual inspection of Hi-C heatmaps in intrachromosomal view with 5 kb resolution. In all cases, the Hi-C heatmap of cells with normal genome (chicken embryonic fibroblasts) [39] was used as a control map to detect abnormal patterns of intrachromosomal interactions. RNA-seq data were obtained from Ulianov et al., 2017 [38] (NCBI GEO accession number GSE76573).

### Fluorescence in situ hybridization

Probes for FISH were labeled with biotin-dUTP (DNA-Synthesis) or digoxigenin-dUTP (Jena Bioscience) by nick-translation, DOP-PCR or PCR with specific primers using chicken genomic DNA or BAC DNA of chicken BAC clone library CHORI-261 (https://bacpacresources.org/chicken261.htm) according to standard protocols [51]. For FISH detection of large clusters of chicken tandem repeats, we used Cy3-labeled oligonucleotide probes [52, 53]. The complete list of probes and analyzed regions is summarized in Additional Table 1. A total of 100–500 ng of indirectly labeled probes was precipitated with a 50 × excess of sheared salmon sperm DNA (ThermoFisher Scientific) or yeast tRNA (Invitrogen) and dissolved in a hybridization mixture containing 50% formamide, 10% dextran sulfate and 2 × SSC. The DOP-PCR-labeled probes were additionally precipitated with a 10 × excess of chicken Cot5 DNA obtained by S1 nuclease digestion of the slowly renaturing fraction of chicken genomic DNA as described earlier [54]. In the case of Cy3-labeled oligonucleotide probes, the concentration of formamide in the hybridization mixture was lowered to 40%. Before hybridization, slides were sequentially treated with 150 µg/ml RNAse A (ThermoFisher Scientific) for 1 h at 37 °C, 0.002% pepsin in 0.01 N HCl for 10 min at 37 °C and postfixed in PBS-buffered 1% PFA for 10 min. Slides were denatured in 2 × SSC containing 70% formamide for 15 min at 70 °C, dehydrated in an ice-cold ethanol series and air-dried. Probes were denatured at 95 °C for 10 min; probe mixes containing chicken Cot5 DNA were further preannealed at 37 °C for 1 h. After denaturation, probes were mounted on slides, sealed with rubber cement and hybridized at 37 °C for 1–2 days in a humid chamber. Posthybridization washes of Cy3-labeled oligonucleotide probes included two changes of 2 × SSC at 40 °C. In all other cases, slides were washed in three changes of 0.2 × SSC at 60 °C followed by two changes in 2 × SSC at 45 °C. Probe detection was carried out as described previously [55]. Briefly, biotin-labeled probes were detected by streptavidin-Alexa 488 (ThermoFisher Scientific) with signal amplification by biotinylated anti-streptavidin antibody (Vector Laboratories) followed by additional incubation with streptavidin-Alexa 488. Digoxigenin-labeled probes were detected with Cy3-conjugated mouse anti-digoxin antibodies (Jackson ImmunoResearch) with signal amplification by Cy3-conjugated anti-mouse secondary antibodies (Jackson ImmunoResearch). After washing, the slides were dehydrated in an ethanol series and mounted in antifade solution (65% glycerol, 2% DABCO) containing 1 μg/ml DAPI.

### Microscopy

Slides were examined under a Leica DM4000 microscope (Leica-Microsystems) using an oil immersion HC PL APO 100 × objective and LAS AF software (Leica-Microsystems) and Leica filter cubes for DAPI (A), Alexa488 (I3) and Cy3 (N2.1) fluorochromes. Images were captured in 1392 × 1040 *.tiff format by a CCD monochrome camera DFC350FX. More than 20 individual metaphase plates were analyzed per slide. Images were assembled in Adobe Photoshop software.

## Results

### TADs and A/B compartment conservation between chicken erythroid progenitor cell line and embryonic fibroblasts

Hi-C libraries of the HD3 erythroid progenitor cell line were obtained from two biological replicates of crosslinked cells. In total, 208 and 211 million paired-end reads were generated from the two biological replicates, respectively. According to the alignment data, more than 90% of the resulting reads were mapped to the chicken genome, with more than 80% of the reads being unique, while 10% of the reads represented PCR duplicates. Less than 7% of the unique reads were mapped within a single restriction fragment, indicating a high enrichment of Hi-C libraries with fragments containing a restriction fragment ligation site.

**Fig. 1:**
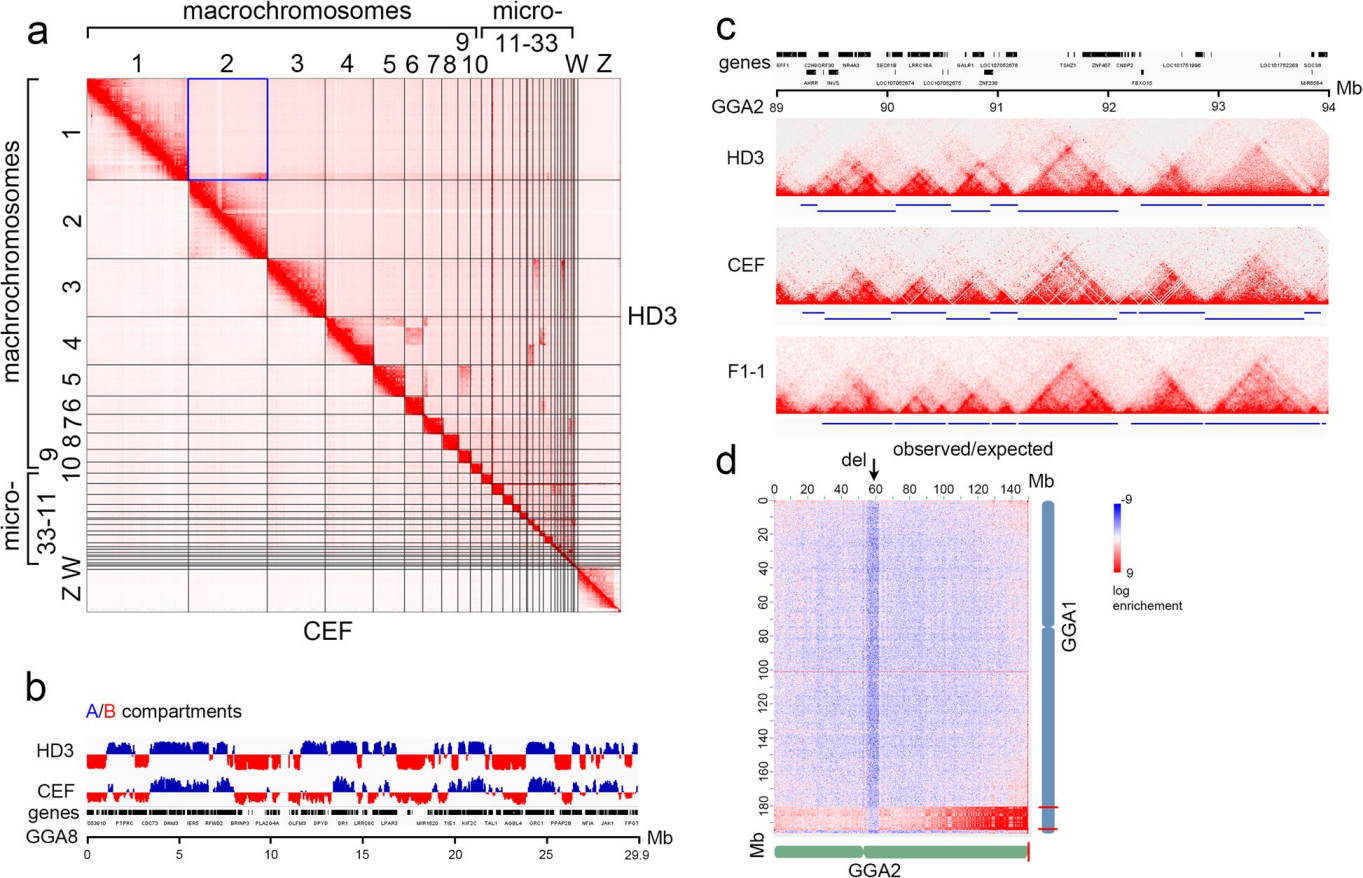
General features of high-throughput chromatin conformation capture (Hi-C) heatmaps of the HD3 chicken cell line. a—Chromosome-scale view of the Hi-C heatmap of genomic contacts in HD3 (upper right) versus chicken embryonic fibroblasts (CEF) (lower left) with a normal genome. Karyotype abnormalities are identified as nondiagonal patterns of enriched interchromosomal interactions; b—Example of A/B compartment profile along GGA8 in HD3 cell line and CEF; c—Example of TAD profile and TAD coordinate calls (blue lines below) in 5 Mb region of GGA2 in HD3 cell line, CEF and chicken granulosa cells F1-1 from preovulatory follicles; d—Enlarged view of HD3 Hi-C heatmap square (a, blue), indicating abnormal enrichment of contacts in the terminal regions of GGA1 and GGA2 as well as significant loss of contacts near the centromere of GGA2. The interaction enrichment gradient is clearly seen along GGA2, starting from GGA2qter. The relative positions of breakpoint regions are shown as red lines on the schematic depictions of chromosomes

Next, we constructed full-genome matrices of chromatin contacts in HD3 cells and visualized them as heatmaps (Fig. 1 a). We measured the *cis/trans* contact ratio in the obtained data to estimate the proportion of noise originating from random ligation of DNA fragments. In both replicates, the *cis/trans* contact ratio varied in the range of 1.6–2.3, which was comparable with our earlier results for chicken embryonic fibroblasts (CEF) [39] and pointed to the low noise and high quality of Hi-C data. The average Pearson correlation coefficient of contacts between the replicates was 0.993, showing good data reproducibility. Valid contacts from both replicates were combined for further study, including examining the three-dimensional chromatin contacts in the HD3 cell line.

Analysis of heatmaps of the HD3 cell line revealed large-scale chromatin domains (**A** and **B **compartments) (Fig. 1 b), and the strength of compartmentalization was generally comparable to that of embryonic fibroblasts. We obtained a genome-wide compartment profile at a resolution of 50 kb and compared the distribution of compartments between HD3 and CEF using published data. As in mammalian cells, compartments in different chicken cell types were only moderately correlated (Pearson correlation 0.55). Some regions that change the compartment status between HD3 and CEF could be seen in the example of GGA8 (Fig. 1 b). Note that in most of the cases, there is a shift from B to A compartments in HD3 cells.

In addition, regions enriched with local contacts, namely, topologically associating domains (TADs), were identified in the genome of chicken HD3 cells (Fig. 1 c). Such contact domains have been detected on both macro- and microchromosomes, similar to previous data for other chicken cell types [39, 48, 56]. In many cases, TADs exhibit a hierarchical structure; that is, large contact-enriched domains can be subdivided into several smaller domains. We also compared topologically associating domains between three chicken cell types: HD3, CEF and granulosa cells of preovulatory follicles. Visual inspection showed that TAD borders appeared conserved between the cell types (Fig. 1 c). Consistent with this, genome-wide TAD calling using the Armatus algorithm showed that 76% of the TAD boundaries identified in CEF coincided with those in HD3.

### Detection of interchromosomal rearrangements in the HD3 cell line

Multiple SVs are usually common for virus-transformed cancer cell lines. Moreover, new SVs frequently appear in immortalized cultures, which have a long history of in vitro propagation. As the chicken HD3 cell line meets both these conditions, we used Hi-C heatmap of HD3 cells to search for large SVs, mainly chromosomal translocations, deletions, and inversions. To verify some translocations identified by the analysis of HD3 Hi-C heatmaps, we used fluorescence in situ hybridization (FISH) with chromosome- and region-specific probes (Additional Table 1).

Visual inspection of the chromosome-sorted whole genome Hi-C map of HD3 cells revealed multiple cases of interchromosomal interactions with elevated frequencies of pairwise contacts compared to the Hi-C map of CEF with normal genome (Fig. 1 a, d). The ratio of interchromosomal contacts to the total number of contacts was two times higher in HD3 cells than in CEF. The translocation breakpoint and orientation of the translocated region can be visually identified based on the gradient of contacts within the region of interchromosomal interactions (Fig. 1 d). We examined Hi-C heatmaps of chromosome pairs and intrachromosomal regions involved in translocation at a 5 kb binning to obtain coordinates of chromosome breakpoints. In total, we mapped 26 large translocations between chromosome pairs, involving chromosome regions ≥ 200 kb (Table 1) and many smaller ones (Additional Table 2). Most macrochromosomes, apart from GGA8 and GGA10, participated in translocation events, for example GGA1: 180,580,000—194,260,000 and GGA2: 149,470,000 (Fig. 1 d, Additional Fig. 1).

**Table 1:** Interchromosomal translocations identified in HD3 cell line based on the analysis of Hi-C heatmaps

Interacting chr1	start, ± 5000 bp	end, ± 5000 bp	Interacting chr2	Insertion site and approximate breakpoint position, ± 5000 bp
1	180,580,000	194,260,000*	**2**	q:149,470,000
4	0*	16,835,000	**5**	qter:59,820,000
4	18,835,000*	51,490,000	**6**	pter:500,000
4	0*	16,835,000	**7**	pter:0–6,310,000
4	51,505,000*	91,280,000	**17**	qter:10,950,000
4	188,350,000	51,490,000*	**19**	qter:9,965,000
5	0*	59,820,000	**9**	ter**
6	500,000	35,460,000	**19**	ter
7	0	6,310,000*	**5**	qter:59,820,000
7	6,145,000	36,905,000	**27**	ter
7	4,110,000	36,905,000	**33**	ter
12	0	19,945,000	**21**	ter
12	0	19,945,000	**23**	qter:5,785,000
13	500,000	18,400,000	**28**	ter
17	505,000*	10,950,000	**28**	ter
17	505,000	10,950,000	**33**	ter
18	0*	11,040,000	**3**	pter:0
21	0	325 000*	**5**	pter:5000
21	0	325 000	**9**	pter:515,000
21	0	6,860,000	**23**	ter
24	0	6,270,000	**3**	p:2,200,000
27	0	5,035,000	**23**	ter
28	0	1,140,000	**12**	ter
28	0	1,140,000	**21**	ter
28	0	1,140,000	**23**	ter
28	0	4,970,000	**33**	ter

^*^ – region orientation relative to acceptor chromosome breakpoint location

^**^ – the breakpoint position on the termini of *p*- or *q*- chromosome arms could not be identified unambiguously on the basis of a interchromosomal gradient pattern on the Hi-C map, the insertion site is therefore marked as terminal region of the chromosome – *ter*

According to HD3 Hi-C heatmap analysis, among macrochromosomes, GGA4 was engaged in translocations with many other chromosomes, namely, GGA5, 6, 7, 17 and 19. For this reason, we focused on a detailed analysis of GGA4 rearrangements by Hi-C and FISH. The intrachromosomal GGA4 contact pattern in HD3 clearly distinguished at least 3 large-scale domains (GGA4p.1, GGA4q.1, GGA4q.2), which were absent in CEF (Additional Fig. 1 a). The borders of these domains coincide with breakpoints on GGA4, while enrichment of contacts within the domain may reflect its insertion in the derivative chromosome and loss of contacts with the remnant GGA4 and/or duplication of the region. However, all GGA4 domains form a significant number of cis-contacts with each other, which suggests the presence of a wild-type GGA4 homologue. In accordance with the Hi-C data, the same GGA4 regions were involved in simultaneous interactions with several chromosomes, which may indicate either heterogeneity of the cell culture with respect to translocation variants or complex translocations that result in derivative chromosome(s) constructed from regions of different chromosomes (Table 1).

**Fig. 2:**
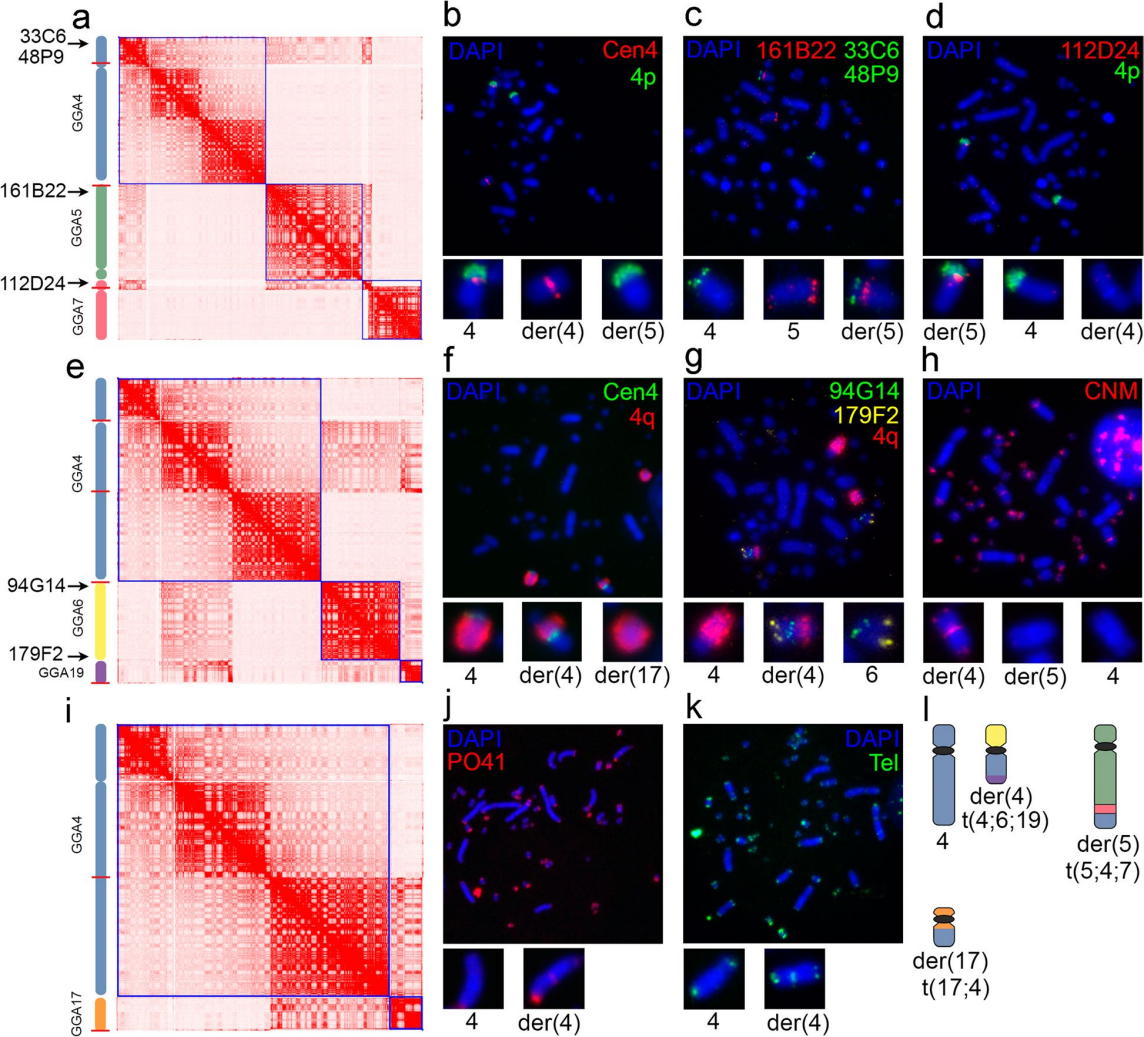
Interchromosomal translocations involving chromosome 4 (GGA4) in the HD3 cell line. a, e, i—Assemblies of HD3 Hi-C heatmaps indicating translocations of different regions of GGA4 to other chromosomes. Enrichment of interchromosomal contacts is detected between GGA4p, GGA5 and a part of GGA7p (a), centromere proximal region of GGA4q, GGA6 and GGA19 (e), centromere distal region of GGA4q with GGA17 (i). Blue squares on the heatmaps represent intrachromosomal contacts (chromosome territories). The relative positions of breakpoint regions are shown as red lines on the schematic depictions of chromosomes. Arrows point to the approximate positions of BAC probes. b, c, d—FISH verification of GGA4p translocations. Loss of GGA4p from one homologue of GGA4 was confirmed by using a GGA4 centromere-specific probe and a GGA4p paint probe (b). Fusion of translocated GGA4p with GGA5 (c) and a part of GGA7p (d) is confirmed by corresponding BAC-based probes. f, g—FISH verification of GGA4q breakage and translocation. Loss of the centromere-distal part of GGA4q from one homologue of GGA4 and its translocation to a microchromosome was confirmed by using a GGA4 centromere-specific probe and GGA4q paint probe (f). Fusion of GGA4 with GGA6 is confirmed by the corresponding BAC-based probes (g). Note the change in the centromere index of derivative chromosome 4 (metacentric) and unstained material in derivative chromosome 4q ter, indicating translocation from another chromosome, presumably GGA19; h, j, k—FISH localization of clusters of chicken tandem repeats CNM (h), PO41 (j) and telomere repeat (Tel) (k) in the HD3 chromosomes. Note the presence of centromeric and terminal clusters of CNM and PO41 repeats and an additional centromeric cluster of telomere repeat in derivative chromosome 4 compared to the normal homologue (h, j, k correspondingly, enlarged images); l—schematic depiction of possible derivative chromosomes containing GGA4 material. Chromosomes were counterstained with DAPI

Based on the identified breakpoints, the gradient of interchromosomal contacts, chromosomes involved and using Juicebox assembly tools [50], we reconstructed possible derivative chromosomes containing parts of GGA4 and verified their occurrence by FISH on metaphase spreads from HD3 cells (Fig. 2, Additional Fig. 1 a’). Apart from normal GGA4, Hi-C analysis revealed the simultaneous interaction of GGA4p.1, GGA5 and GGA7p (Fig. 2 a). By using a paint probe to the short arm of GGA4 together with a GGA4 centromere-specific probe, we showed the presence of a normal homologue of GGA4 with an intact centromere and short arm, translocation of the short arm to other similarly sized macrochromosomes and a complete loss of a normal short arm from the other homologue of GGA4. Derivative GGA4 became metacentric with a gain in the short arm of submetacentric GGA4 (Fig. 2 b).

With probes to the terminal region of GGA5q, we confirmed that GGA4p translocated to GGA5 and clarified the breakpoint position on GGA5qter (Fig. 2 c). By using probes specific to GGA7, we also verified translocation of a part of the short arm of GGA7 to derivative GGA5 (Fig. 2 d). Note that this region is lost from both of the homologs of GGA7 (Additional Fig. 1 a’’), which is also seen in the Hi-C map of GGA7 as a drastic depletion of interactions in this region with the rest of GGA7 (Fig. 2 a).

As followed from the Hi-C map, the other breakpoint separated centromere-proximal (GGA4q.1) and centromere-distal (GGA4q.2) regions of the long arm of GGA4 (Fig. 2 e, i). The approximate breakpoint position was confirmed by FISH with probes flanking the breakpoint region (Additional Fig. 1 a-a’). GGA4q.1 was involved in interactions with GGA6 and GGA19 (Fig. 2 e), while GGA4q.2 was involved in interactions with chromosome 17 (Fig. 2 i). FISH signals from the paint probe to GGA4q were observed on three chromosomes: seemingly normal GGA4 with intact centromere, derivative metacentric GGA4 and on an unidentified microchromosome, likely chromosome 17 (Fig. 2 f).

By using probes for GGA6, we confirmed that the translocation of GGA6 on GGA4q.1 resulted in the formation of the metacentric derivative GGA4 (Fig. 2 g). In the centromere region of the metacentric derivative GGA4, we detected clusters of CNM repeats characteristic of the centromere region of GGA6, as well as telomere repeat (Fig. 2 h, k). Additional clusters of CNM and PO41 repeats, usually located in centromeric and terminal regions of acrocentric microchromosomes [52, 53], were also observed in the terminal regions of derivative GGA4 (Fig. 2 h, j), which may indirectly indicate translocation of the microchromosome, presumably GGA19, as followed from the Hi-C map (Fig. 2 e). However, the identity of the translocated microchromosome was not tested by FISH. In summary, GGA4 rearrangements identified by Hi-C and FISH are in good agreement and show the presence of at least three derivative chromosomes containing different regions of GGA4 in the HD3 karyotype (Fig. 2 l).

Additionally, we confirmed the translocation of the 10 Mb region from the long arm of GGA1 to the terminal region of the long arm of GGA2 by using BAC clone-based probes (Additional Fig. 1 b-b’). FISH signals from BAC probes to the translocated region of GGA1 and terminal region of GGA2 were observed in the derivative GGA2. Interestingly, the translocated region was not lost from either homolog of GGA1, thus supporting translocated duplication of the GGA1 segment. In the terminal region of the long arm of GGA2 containing the translocated region, we also observed additional clusters of PO41 repeat and a new cluster of CNM repeat (Additional Fig. 1 b’’-b’’’), possibly associated with the identified translocation. As a result of the translocation, as in the case of GGA4, GGA2 homologs became heteromorphic (Additional Fig. 1 b’’’’).

We further focused on abnormal interchromosomal contacts of microchromosomes with the exception of NOR-bearing GGA16 and the smallest microchromosomes GGA30-32 due to the low total number of uniquely mapped reads and poor genome assembly of these chromosomes. Also GGA29, 34–38 were not analyzed as they are missing in the GalGal5 chicken genome assembly. Of the 18 analyzed microchromosomes, 11 formed anomalous pairwise contacts with other microchromosomes and/or macrochromosomes (Table 1). Some microchromosomes exhibited multiway contacts with more than one chromosome, an example of such interactions being those of microchromosome 21, with an identified breakpoint at 325 kb (Additional Fig. 1 c–c’). The terminal region of GGA21 translocated to GGA5 and GGA9, while GGA21 also showed enrichment of contacts with microchromosomes 12 and 23 as well as a part of microchromosome 28. FISH with a probe to the terminal region of GGA21 detected at least three heteromorphic chromosomes in the HD3 karyotype, the largest of which was another derivative of GGA5 (Additional Fig. 1 c’’-c’’’). Interestingly, in most cases, we could not detect the position of the breakpoint within the microchromosomes, with the exception of microchromosomes GGA21 and GGA28, because enrichment of interactions was observed on the entire chromosome. We hypothesize that breakpoints may occur in the termini of microchromosomes, possibly within regions rich in tandemly repeated sequences that are poorly represented in genome assemblies and, therefore, usually absent from Hi-C maps. Chicken microchromosomes are acrocentric, so we could not rule out that at least some breakpoints could be associated with centromere regions, as in Robertsonian translocations. Collectively, most translocations involving microchromosomes resembled “terminal fusions'' and could not be classified precisely. In addition to the inability to map breakpoints in microchromosomes, in some cases, we were also unable to discriminate the directionality of the interchromosomal interaction gradient to identify the correct orientation of the microchromosome participating in the “fusion” events.

Based on the Hi-C interaction pattern and chromosome-based assembly, we estimated the chromosome number in the HD3 cell line to be about 48 chromosomes, compared to 2n = 58 for GalGal5. The severe aneuploidy of HD3 cell line was confirmed by the analysis of DAPI-stained metaphase spreads, where the median number of chromosomes counted 2n = 55 ± 1.5, compared to 2n = 78 for normal chicken karyotype (Additional fig. 2).

Furthermore, we applied the HiCtrans tool with default parameters [23] for the automatic search for large translocated regions and breakpoints. In our set of 26 pairs of interacting chromosomes containing regions with increased Hi-C counts (Table 1), HiCtrans found 894 translocation boxes, half of which were located within 2 bins from the supposed breakpoint. The other half of translocation boxes was found within the patterns of increased interactions, suggesting false-positive detection of the pattern borders. It is worth noting that HiCtrans is not suitable to discriminate between interchromosomal interactions caused by errors in genome assembly and real interchromosomal translocations. Generally, the number of detected translocation boxes with a varying ratio of true border/false-positive boxes correlated with the size of translocation. For example, more than 100 boxes were found in t(1; 2), t(4; 6), t(5; 9) and t(7; 5). On the other hand, the HiCtrans algorithm could not verify some translocations with microchromosomes, especially those with “terminal fusions”. In total, visual analysis of HD3 Hi-C heatmaps provided more reliable results than computational algorithms. Further development of new algorithms to detect translocations would be useful for better and more versatile performance on the particular Hi-C dataset.

### Detection of intrachromosomal rearrangements in the HD3 cell line

**Fig. 3:**
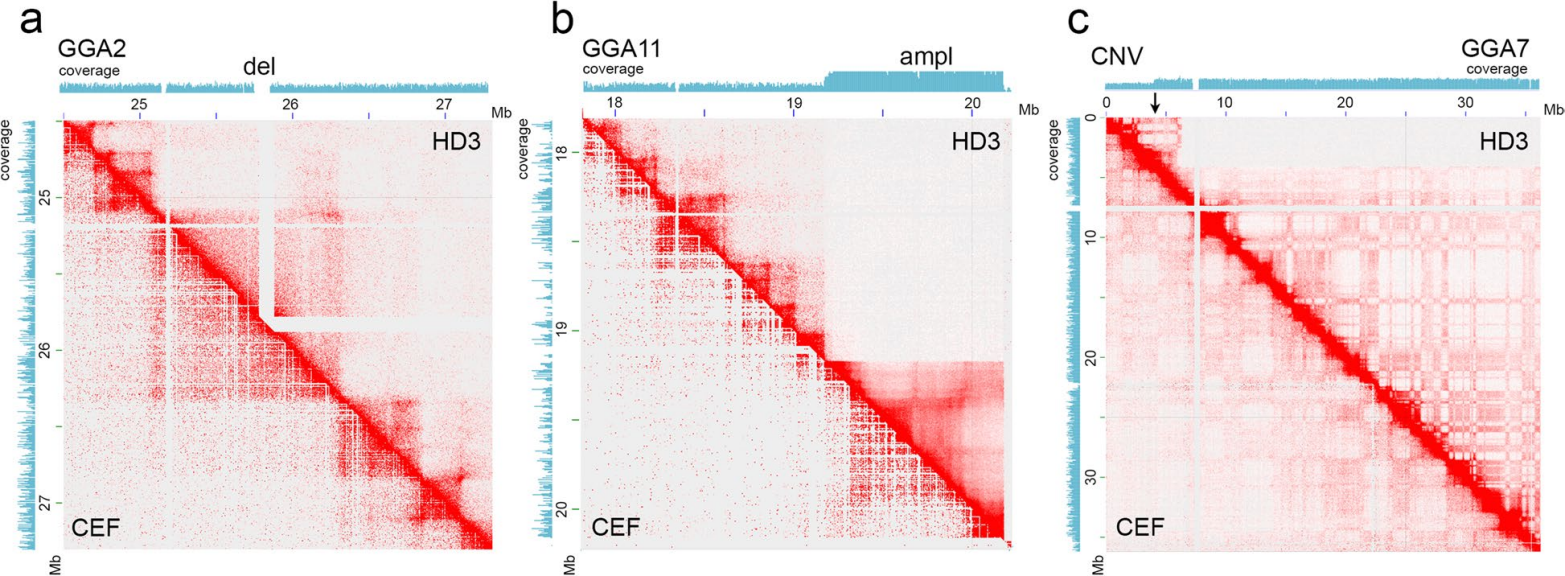
Intrachromosomal structural variations in the HD3 cell line. a-c—View of the Hi-C heatmap of genomic contacts in HD3 (upper right) versus CEF (lower left) with a normal genome. Genome read coverage tracks are shown. a—example of deletion (del) of the 20 kb region in GGA2; b—amplification (ampl) of the ~ 1 Mb terminal region on GGA11; c—copy number variation (CNV) within a region of GGA7p. The arrow points to the position of the breakpoint associated with the translocation of a part of GGA7p to GGA33

Intrachromosomal SVs are also highlighted on Hi-C heatmaps by the change in the pattern (intensity and distribution) of long-range *cis* interactions. However, it should be noted that the identification of significant SVs could be blurred by shortcomings of the reference genome assembly. When visually identifying intrachromosomal SVs, we used the Hi-C heatmap for CEF as a reference map and considered only those anomalous patterns that were not expressed in CEF (Fig. 3 a-c). In addition, we analyzed normalized read coverage along chromosomes to identify possible large-scale duplications of chromosomal regions, including the previously mentioned duplication of translocated qter of GGA1 (Additional Fig. 1 b-b’) and amplification in the q-terminal region of GGA11 (Fig. 3 b). Increased coverage was observed for chromosome W, which is expected to be in monosomic (ZW) condition, as the HD3 cell line was generated from female cells. FISH with probes to sex chromosomes confirmed that in the HD3 karyotype, disomy of the W sex chromosome is found in the presence of the Z chromosome (Additional Fig. 3). Generally, we identified only a subtle number of intrachromosomal SVs compared to interchromosomal ones, which are summarized in Additional Table 3. Most SVs were classified as deletions. Significant coverage loss in the GGA2: 54 325 000—61 685 000 region was possibly associated with the deletion of this region in one homolog of GGA2 (Fig. 1 d). In some cases, loss of cis-contacts or deletions of chromosomal regions were connected with certain intrachromosomal translocations. In translocation t(24; 3), region GGA3: 0—2 195 000 is devoid of interactions with GGA24, while in t(18; 3), it remains intact on GGA3. It is possible that two homologs of GGA3 underwent independent translocations: one with GGA18 and another with GGA24. Alternatively, the population of HD3 cells is heterologous relative to these translocations, and different subclones bear certain types of derivative GGA3. The copy number variation in more than half of GGA7p coincides with the region missed during t(7; 33) (Fig. 3 c). However, the same region is preserved on derivative chromosome 5 t(5;4;7), which may indicate that both homologs of GGA7 are involved in translocation and/or that translocation and deletion of a part of GGA7p are independent events. A low number of large intrachromosomal SVs could be a specific feature of HD3 cells; however, many smaller-scale SVs at the level of TADs could escape visual detection by Hi-C and require aid from other whole-genome sequencing approaches.

Persistent abnormal long-range intrachromosomal interactions identified in Hi-C maps, for example, on GGA1, were revealed in cancerous cell lines HD3 and DT40 [56] and in cells with a normal genome (CEF) (Additional Fig. 4). We classify these regions as misplaced contigs against the reference GalGal5 version of the chicken genome. In Hi-C maps of follicular cells, where reads were mapped to GalGal6, we did not find these abnormal interactions. This again demonstrated the need for cell type comparisons to differentiate between real SVs and assembly errors in species with still improving genome assemblies.

## Discussion

### Domain conservation between cell types

Hi-C, being primarily a method of high-throughput analysis of the three-dimensional genome topology of interphase nuclei, has recently expanded into the detection of karyotype rearrangements in cancers and transformed cell lines [5, 18]. This is particularly beneficial in studies involving cancer cells, as it allows simultaneous assessment of structural and functional genomic domains, such as TADs and compartments, together with various SVs and large-scale karyotype abnormalities. In our study, we obtained Hi-C profiles of TADs and A/B compartments for the chicken erythroid HD3 cell line. Notably, in the HD3 cell line, which is generated from erythroid precursors, the genome is organized into TADs and subTADs with hierarchical structure. In contrast, nucleated erythrocytes from various vertebrate species, including chicken, do not possess TADs and loops typical for other somatic cell types [39, 57]. The conventional genome topology is therefore maintained in HD3 cells, which are immortalized in the early erythroid differentiation stage (BFU/CFU) [29]. We believe that early chicken erythroid commitment may resemble that of mammals and is not accompanied by dramatic changes in the 3D genome organization in erythroid progenitor cells. It has recently been shown that TADs and A/B compartments are clearly distinguishable in Hi-C heatmaps of isolated mouse fetal liver erythroid precursors before stimulation of terminal differentiation with erythropoietin [58] and in human primary fetal and adult CD34 + erythroblasts until at least day 11 of maturation in vitro [59]. Chicken erythrocyte-specific loss of TADs and mammalian erythrocyte nuclear condensation and extrusion occur at later stages of terminal erythroid differentiation.

Comparative analysis of computationally called TAD borders between HD3 cells and CEF showed a significant overlap in TAD coordinates. Mammalian TADs are commonly considered to be generally conserved between cell types, indicating preservation of regulatory landscapes in particular TADs [4, 60, 61]. Recent evidence from the analysis of TAD profiles in dozens of cells of different origins, including primary tissues and cancer cell lines, suggests that a large number of subTADs are cell type specific, and only a subtle number of TADs have conserved borders in many studied cell types [62]. However, our visual comparison of Hi-C heatmaps between chicken HD3 erythroblasts and CEF revealed only a limited number TADs/subTADs or looping interactions within them differed between the two cell types (Additional Fig. 5 a, b). At the same time, we cannot rule out that a greater diversity in the 3D organization of chromatin between CEF and HD3 cells will be discernible when higher-resolution Hi-C heatmaps are obtained.

Chromosomal rearrangements observed in HD3 cell line may also lead to changes in local chromatin interactions within TADs, as in the duplicated region (Additional Fig. 5 c), and to reshuffling of TADs near the translocation breakpoint (Additional Fig. 5 d). Nevertheless, in many cases interactions within TAD remain largely undisturbed by the rearrangements, as shown for a small deletion within TAD on GGA2 (Fig. 3 a). Compartments, in turn, are nonconserved features and vary significantly among cell types, following changes in the epigenetic status of a particular genomic region [60, 63].

### Karyotype rearrangements in different chicken cell lines

In our study, we showed the potential of Hi-C in predicting chromosome rearrangements in cell lines from chicken, such as the virus-transformed erythroblastoid cell line HD3. As for many human cancer cell lines, karyotype abnormalities have been established for a wide range of chicken continuous cell lines derived from virus-transformed cells, spontaneously transformed cells and tumors. However, these data in continuous chicken cell lines are fragmentary and generally limited to the analysis of numerical karyotype abnormalities, such as chromosomal aneuploidies, by classical cytogenetic approaches using Giemsa-stained metaphase spread chromosome preparations and FISH with chromosome-specific probes. Significant deviations from diploid chromosome number have been demonstrated for the hepatocellular carcinoma cell line LMH [64], avian leukosis cell line LSCC–H32 and CEC-32 cell line [65], several cell lines derived from Marek’s disease T-cell lymphomas, such as HPRS Line 1 and HPRS Line 2 [66], ALV-J-induced fibrosarcoma cell line [67] and others. In many tumor-derived cell lines, chromosomal heteromorphism involving one or several pairs of macrochromosomes has been demonstrated, including amplification of the short arm of chromosome 1 (1p +) in different cell lines from Marek’s disease lymphomas [68], the heteromorphic chromosome 1 pair in MSB-1, marker chromosomes derived from rearrangements involving chromosome 3 or chromosome 5 and unidentified elements in HPRS Lines 1 and 2 [66].

The application of a particular cell line and its use as a model system in biomedical research may be compromised by the degree of chromosome structural variations and their implications on genome functionality, cell phenotypes and in vitro propagation. Decreasing sequencing costs and constantly updating chromosome-level chicken genome assembly make possible a more detailed analysis of chromosome SVs with higher resolution compared to cytogenetic tools. To date, however, the chicken bursal lymphoma B-cell line DT40 is one of the few examples in a wide list of chicken cell lines for which chromosome composition has been investigated by both cytogenetic [41] and genomic approaches [56, 69]. DT40 has a relatively normal karyotype with duplications in macrochromosome GGA2 [41, 69] and microchromosomes GGA14, GGA20 and GGA24 [69]. Analysis of DT40 Hi-C maps showed small translocation insertion of three loci, *MYC*, *SOX5*, and *MIR221*, on a single integration site on GGA1 and two intrachromosomal translocations, one between GGA14 and GGA24 and one between GGA20 and GGA23 [56].

Our analysis of Hi-C maps has demonstrated that numerous interchromosomal translocations are common in the HD3 cell line, leading to an extensively rearranged karyotype, where most of the chromosomes participate in translocation events. Several translocations, including those resulting in the formation of der(2), der(4), der(5), der(21), were confirmed by FISH with chromosome-specific probes and showed good agreement between the two approaches for chromosome translocation mapping. The mentioned derivative chromosomes were in a heterozygous state in the HD3 karyotype with either normal or differently rearranged homologous chromosomes. By a more detailed analysis of GGA4 in the HD3 karyotype, we noticed that GGA4 was prone to breakage, as had been shown earlier in the DT40 cell line [41]. One of the breakpoints on GGA4, resulting in translocation of GGA4p, was located near the centromere region. Interestingly, GGA4 has been derived from the fusion of ancestral bird chromosome 10 (GGA4p) and chromosome 4 [42, 43, 70, 71]. Breakpoints in GGA6 and GGA9 were also located near the centromere gap in the genome assembly. Among UV-induced chicken chromosome rearrangements, there was at least one case of reciprocal translocation between an unidentified microchromosome and a part of the long arm of GGA1 where a break occurred at the centromere region of the microchromosome [72].

It is possible that chromosome instability and cell line-specific chromosome breakpoints may be associated with certain “fragile” chromosomal regions. We noted that in most microchromosomes, translocation breakpoints were located at the terminal regions of chromosomes, and the pattern of their interactions on Hi-C maps testified to unbalanced translocations, resembling “chromosomal fusions”. Fusions of chromosomes 10, 16, 28 and W were previously identified in the fibroblast-like cell line DF1 [73]. In Hi-C maps of DT40 cells, novel adjacencies between chromosome pairs GGA14-GGA24 and GGA20-GGA23 appeared at the chromosome ends. “Chromosomal fusions'' could be a frequent mode of microchromosomal translocations during chicken karyotype rearrangements. In chicken genome assembly, chromosome ends of acrocentric microchromosomes are usually poorly assembled due to the large number of repeats. At the same time, it has recently been shown that many de novo annotated genes are embedded in the terminal regions of chicken chromosomes [74]. Thus, breakpoints located in the terminal regions of chromosomes can be reanalyzed when improved chicken chromosome assemblies are available.

Chromosomal rearrangements, such as multiple copy number variations, may influence the gene expression profile and limit the usage of HD3 as a model of erythroid differentiation. For example, ~ 2 Mb partial deletion of the gene-rich region in GGA1 comprises the β-globin gene (*HBG2*). However, we noticed that the most important genes involved in erythropoiesis and differentially expressed in HD3 cells, such as *GATA2*, *Tal1*, *ZFPM1*, *LMO2*, *NFE2L2*, *LDB1*, *KLF-4*, *FECH*, *CPOX*, *TFRC*, *H1F0*, *CA2* and α-globin gene π (*HBZ*), were not disrupted by SVs. Thus, the HD3 cell line can be applied to explore the relationship between genome structure and function, as well as the effect of genomic rearrangements on gene expression.

## Conclusions

Here, we obtained a Hi-C chromatin contact map for chicken erythroid progenitor cells (HD3 cell line). In the HD3 cell line, in contrast to mature and immature erythrocytes, the interphase genome is organized into distinct topologically associating domains. TAD borders were generally conserved between HD3 and other chicken cell types, while A/B compartments were only moderately correlated. Analysis of contact patterns in the Hi-C heatmaps showed that the HD3 chicken cell line has a highly rearranged karyotype, with most of the chromosomes involved in unbalanced translocations. Several of the identified chromosomal translocations were confirmed by FISH. To our knowledge, the HD3 cell line is the first example of a continuous chicken cell line with such a drastically rearranged karyotype and can be used to study general mechanisms leading to karyotype instability and chromosomal diversity.

## Supplementary Information

**Additional file 1.**

## Data Availability

The datasets generated and analyzed during the current study have been deposited in NCBI's Gene Expression Omnibus [75] and are accessible through GEO Series accession number GSE217327.

## Abbreviations

3D: Three-dimensional
AEV: Avian erythroblastosis virus
BAC: Bacterial artificial chromosome
BFU/CFU: Burst-forming unit erythroid / colony-forming unit erythroid
C-methods: Chromosome conformation capture methods
4C-seq: Circular chromosome conformation capture
5C: Chromosome conformation capture carbon copy
CEF: Chicken embryonic fibroblasts
FISH: Fluorescence in situ hybridization
GGA: *Gallus gallus domesticus* Chromosome
Hi-C: High-throughput chromosome conformation capture
PFA: Paraformaldehyde
SVs: Chromosome structural variations
TADs: Topologically associating domains
