# Supplementary material for: Hi-C analysis of genomic contacts revealed karyotype abnormalities in chicken HD3 cell line
Source: BMC Genomics. 2023 Feb 7;24:66. doi: 10.1186/s12864-023-09158-y (PMC9906895; doi:10.1186/s12864-023-09158-y)
Supplement: Supplementary file 1 — Additional file 1. [file 12864_2023_9158_MOESM1_ESM.pdf]

Supplementary Material

Hi-C analysis of genomic contacts revealed karyotype abnormalities in chicken HD3 cell line

Maslova A.[1], Plotnikov V.[1], Nuriddinov M.[2], Gridina M.[2], Fishman V.[2], Krasikova A.[1]*

[1] Saint-Petersburg State University, Saint-Petersburg, Russia

[2] Institute of Cytology and Genetics SB RAS, Novosibirsk, Russia

Corresponding author: a.krasikova@spbu.ru

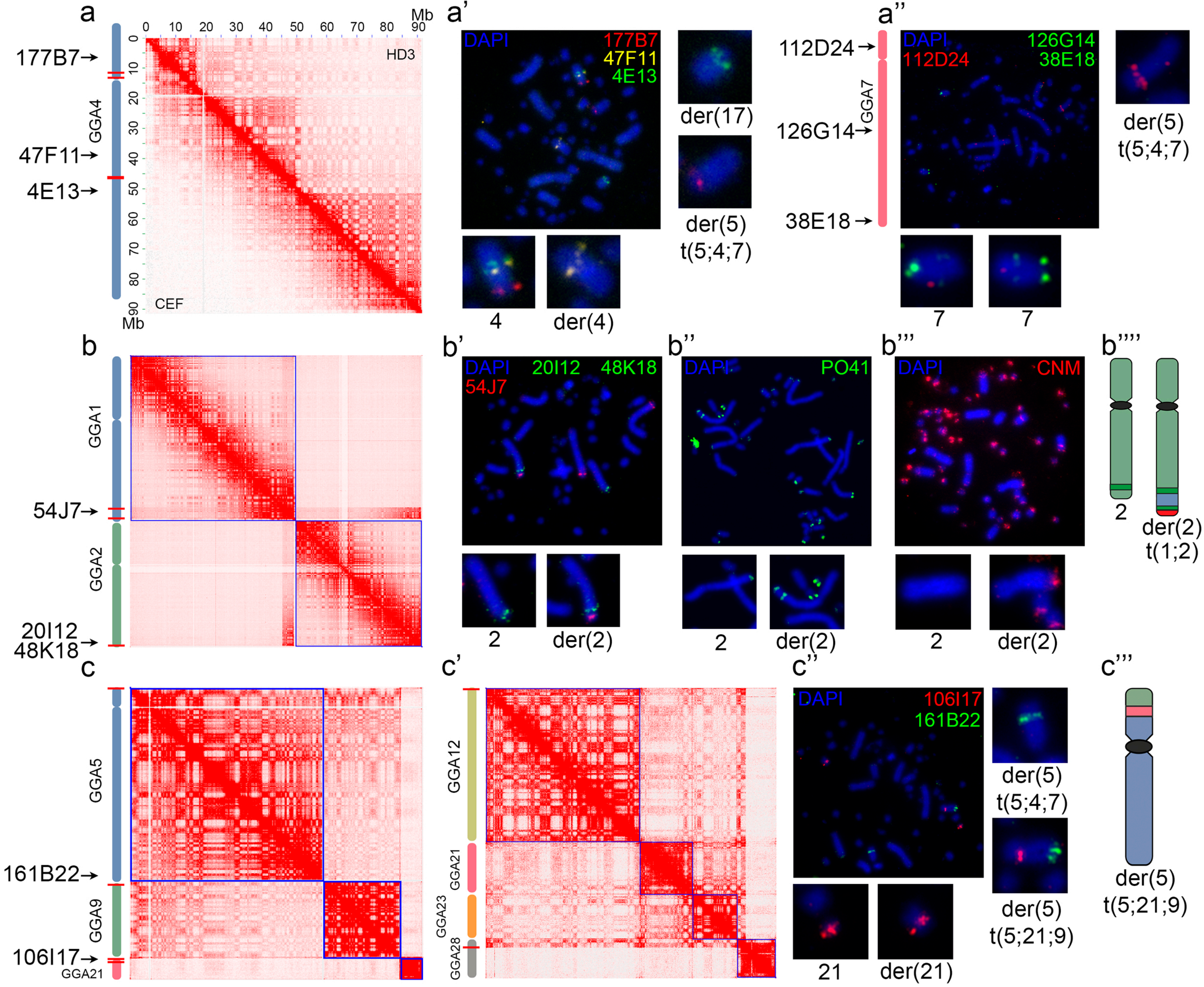

a
177B7→
GGA4
47F11→
4E13→
Mb
HD3
CEF
a'
DAPI
177B7
47F11
4E13
der(17)
der(5)
t(5;4;7)
4
der(4)
a''
112D24→
GGA7
126G14→
38E18→
DAPI
112D24
126G14
38E18
der(5)
t(5;4;7)
7
7
b
GGA1
54J7→
GGA2
20I12
48K18→
b'
DAPI
20I12
48K18
54J7
2
der(2)
b''
DAPI
PO41
2
der(2)
b'''
DAPI
CNM
2
der(2)
b''''
2
der(2)
t(1;2)
c
GGA5
161B22→
GGA9
106I17→
GGA21
c'
GGA12
GGA21
GGA23
GGA28
c''
DAPI
106I17
161B22
der(5)
t(5;4;7)
der(5)
t(5;21;9)
21
der(21)
c'''
der(5)
t(5;21;9)

Additional Figure 1. Interchromosomal rearrangements in the chicken HD3 cell line involving macro- and microchromosomes. a, b, c, c' - assemblies of the HD3 Hi-C heatmaps, indicating intrachromosomal contacts of GGA4 in HD3 cells versus chicken embryonic fibroblasts (CEF) (**a**), translocation between GGA1 and GGA2 (**b**), translocations between macro- and microchromosomes (**c**, **c'**). Blue squares on the heatmaps represent intrachromosomal contacts (chromosome territories). Relative positions of breakpoints are shown as red lines on the schematic depictions of chromosomes. Arrows point to the approximate positions of BAC-probes. **a'-a'** – 3-color FISH verification of breakage of one of the homologs of GGA4 on metaphase spreads of HD3. **a''** – FISH verification of translocation of a part of GGA7p and a deletion of this fragment from both homologs of GGA7 on metaphase spreads of HD3 using BAC-based probes. **b'-b'''** – FISH verification of translocation between GGA1 and GGA2 on metaphase spreads of HD3 using BAC-based probes (**b'**) and detection of new clusters of PO41 repeats (**b''**) and CNM repeats (**b'''**) on the derivate homolog of GGA2 with repeat-specific probes. **b''''** – schematic depiction of normal and derivative homologs of GGA2. **c''** – FISH verification of translocation between GGA5 and GGA21 on metaphase spreads of HD3. Note the trisomy of the translocated region in the short arm of GGA21 and different size of chromosomes containing material of GGA21 (enlarged images). **c'''** – schematic depiction of derivative GGA5 with t(5;21;9). Chromosomes are counterstained with DAPI.

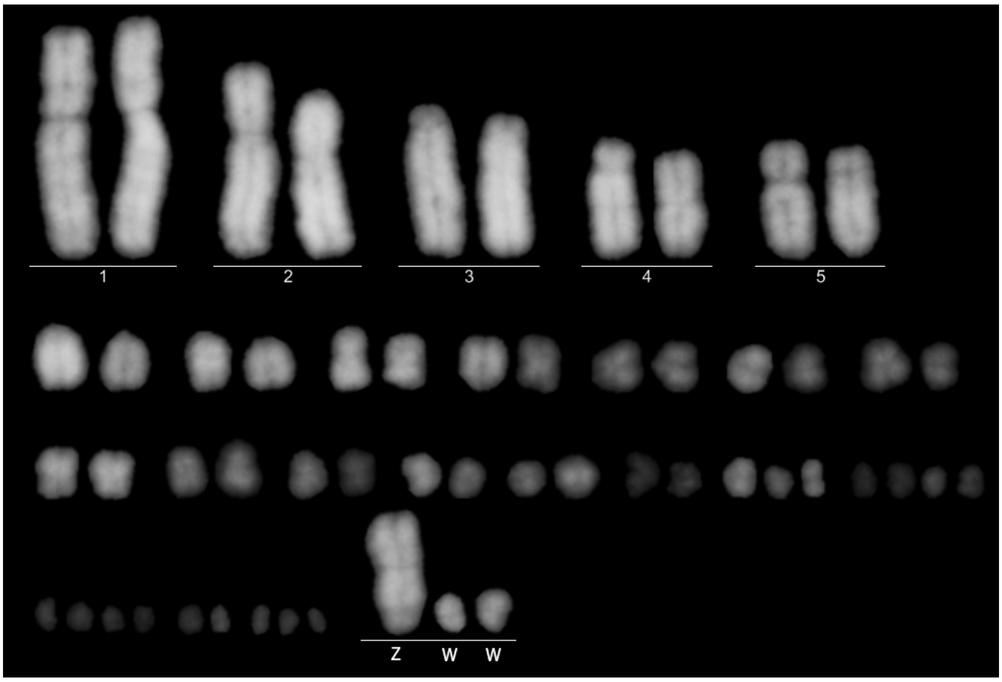

Additional Figure 2. The representative karyotype of the HD3 cell line. Chromosomes are lined up according to their size. Identified chromosomes are numbered. Chromosomes are counterstained with DAPI.

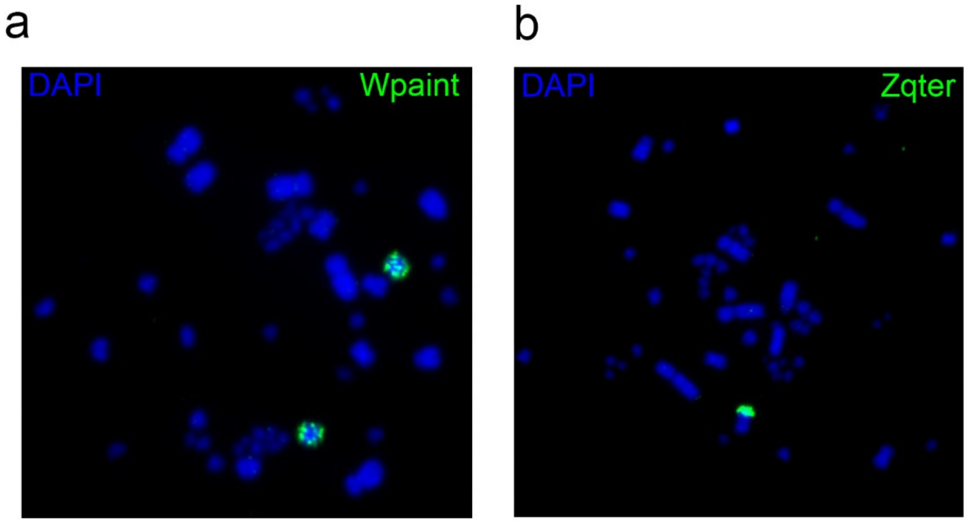

Additional Figure 3. Disomy of the W sex chromosome in the presence of the Z chromosome in the HD3 karyotype. FISH with W chromosome paint (**a**) and microdissected q-terminal heterochromatic region of GGAZ (**b**) as probes. Chromosomes are counterstained with DAPI.

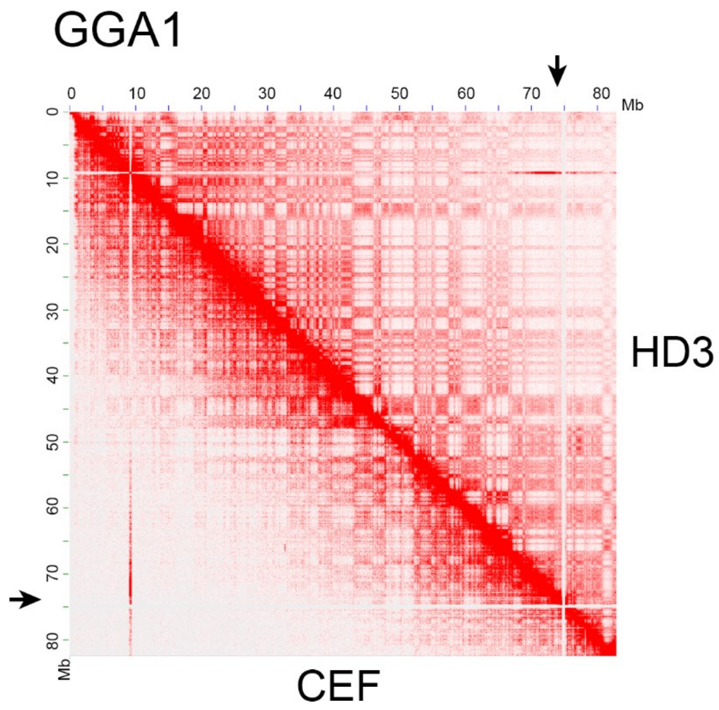

Additional Figure 4. Abnormal intrachromosomal contacts caused by genome assembly errors in GalGal5. View of the Hi-C heatmap of genomic contacts in the HD3 cell line (upper right) versus chicken embryonic fibroblasts (CEF) (lower left) with a normal karyotype. Arrow points to the increased number of genomic contacts near the SEMA3A and LOC107053979 loci observed in both HD3 and CEF.

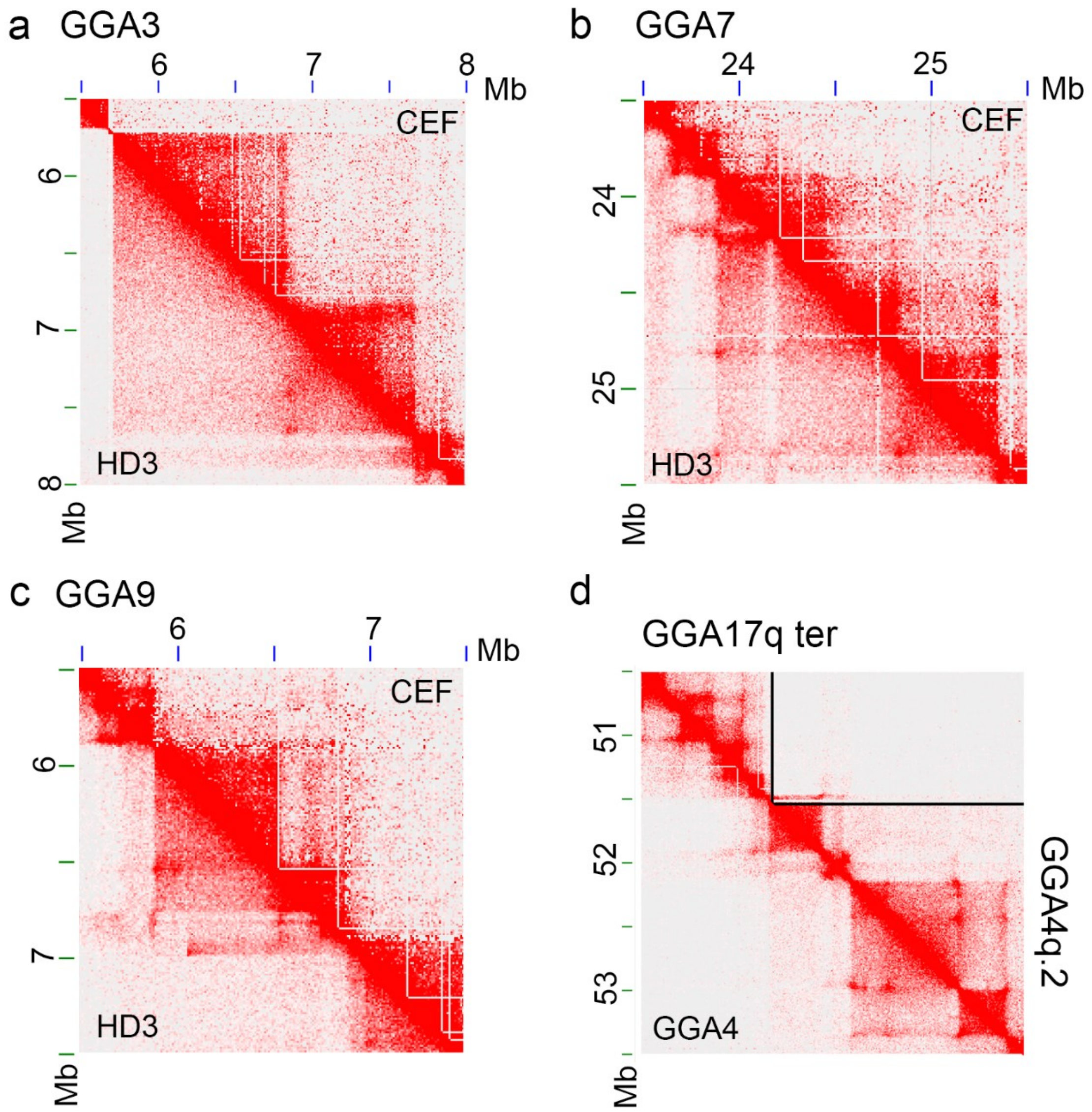

Additional Figure 5. Changes in the TADs (sub)structure associated with cell type (a, b) or chromosome rearrangements (c, d). a – View of the Hi-C heatmap of ~6-8 Mb of GGA3 in HD3 (lower left) versus CEF (upper right); note the absence of the upper TAD in HD3 cells. **b** – View of the Hi-C heatmap of ~23.5-25.5 Mb of GGA7 in HD3 (lower left) versus CEF (upper right); note the change within the domain structure and additional loop domains in HD3 cells. **c** – View of the Hi-C heatmap of ~6-7 Mb of GGA9 in HD3 cell line (lower left) versus chicken embryonic fibroblasts (CEF) (upper right); note the change within the domain structure due to duplication of 6.07-6.99 Mb in HD3 cells. **d** – Assembly of the HD3 Hi-C heatmaps, indicating translocation between GGA17qter and GGA4q.2 with a breakpoint at 51.505 Mb (upper right, black lines separate chromosomes) versus GGA4 homologue (lower left). Note the appearance of neo-TAD between GGA17qter and GGA4q.2 crossing the breakpoint.

Additional Table 1. List of FISH probes used for verification of translocations identified by Hi-C in the chicken HD3 cell line.

Probe ID	Chromosomal localization	Probe origin	References
54J7	GGA1: 185647688-185877592	BAC-clone	https://bacpacresources.org/chicken261.htm
48K18	GGA2qter*	BAC-clone	
20I12	GGA2qter*	BAC-clone	
177B7	GGA4: 5725062-5925483	BAC-clone	
33C6	GGA4: 6419141-6610711	BAC-clone	
48P9	GGA4: 7108351-7324973	BAC-clone	
47F11	GGA4: 41547668-41747405	BAC-clone	
4E13	GGA4: 52490280-52709710	BAC-clone	
161B22	GGA5: 57365803-57524533	BAC-clone	
94G14	GGA6: 811765-995465	BAC-clone	
179F2	GGA6: 35037508-35206756	BAC-clone	
112D24	GGA7: 121180-292753	BAC-clone	
126G14	GGA7: 13125892-13385143	BAC-clone	
38E18	GGA7: 36600879-36791726	BAC-clone	
106I17	GGA21: 135847-355071	BAC-clone	
Cen4	Centromere region of GGA4	PCR with specific primers from Cen4 reference sequence AB556725	Shang et al., 2010; Krasikova et al., 2012
4p	GGA4p	Needle-based microdissection of GGA4p	Maslova et al., 2015
4q	GGA4q	Needle-based microdissection of GGA4q	Maslova et al., 2015
Wpaint	GGAW	Needle-based microdissection of GGAW	
Zqter	q-terminal heterochromatic region of GGAZ	Needle-based microdissection of GGAZqter	
CNM	Centromere and pericentromere regions of microchromosomes; centromere regions of GGA6, GGA9, two clusters on GGA3q, GGAWp	Oligonucleotide	Matzke et al., 1990; Krasikova et al., 2006
PO41	Subtelomere and pericentromere regions of microchromosomes; subtelomere regions of GGA1p, GGA1q, GGA2q, GGAWp, and GGAZp	Oligonucleotide	Wicker et al., 2005; Deryusheva et al., 2007
Tel	Terminal regions of all chromosomes;<br>interstitial sites on GGA1, GGA2q, GGA3q, and GGAW	Oligonucleotide	Meyne et al., 1989; Nanda and Schmid, 1994

* - the position of these CHORI-261 BAC clones was specified by FISH on metaphase chromosomes of chicken embryonic fibroblasts (CEF). In GalGal5 chicken genome assembly 48K18 and 20I12 were erroneously placed on GGA14: 12868028-13032026 and GGA14: 13386106-13599690 correspondingly.

References

1. Shang W-H, Hori T, Toyoda A, Kato J, Popendorf K, Sakakibara Y, et al. Chickens possess centromeres with both extended tandem repeats and short non-tandem-repetitive sequences. Genome Res. 2010;20:1219–28.

2. Krasikova A, Fukagawa T, Zlotina A. High-resolution mapping and transcriptional activity analysis of chicken centromere sequences on giant lampbrush chromosomes. Chromosome Res. 2012;20:995–1008.

3. Maslova A, Zlotina A, Kosyakova N, Sidorova M, Krasikova A. Three-dimensional architecture of tandem repeats in chicken interphase nucleus. Chromosome Res. 2015;23:625–39.

4. Matzke MA, Varga F, Berger H, Schernthaner J, Schweizer D, Mayr B, et al. A 41–42 bp tandemly repeated sequence isolated from nuclear envelopes of chicken erythrocytes is located predominantly on microchromosomes. Chromosoma. 1990;99:131–7.

5. Krasikova A, Deryusheva S, Galkina S, Kurganova A, Evteev A, Gaginskaya E. On the positions of centromeres in chicken lampbrush chromosomes. Chromosome Res. 2006;14:777–89.

6. Wicker T, Robertson JS, Schulze SR, Feltus FA, Magrini V, Morrison JA, et al. The repetitive landscape of the chicken genome. Genome Res. 2005;15(1):126-36.

7. Deryusheva S, Krasikova A, Kulikova T, Gaginskaya E. Tandem 41-bp repeats in chicken and Japanese quail genomes: FISH mapping and transcription analysis on lampbrush chromosomes. Chromosoma. 2007;116:519–30.

8. Meyne J, Ratliff RL, MoYzIs RK. Conservation of the human telomere sequence (TTAGGG) among vertebrates. Proc Natl Acad Sci USA. 1989;86(18):7049-53.

9. Nanda I, Schmid M. Localization of the telomeric $(TTAGGG)_n$ sequence in chicken *(Gallus domesticus*) chromosomes. Cytogenet Genome Res. 1994;65:190–3.

Additional Table 2. Small interchromosomal translocations identified in the HD3 cell line based on the analysis of Hi-C heatmaps.

Interacting chr1	start, ± 5000 bp	end, ± 5000 bp	Interacting chr2	Insertion site / approximate breakpoint position, ± 5000 bp
**1**	196140000	196200000	**2**	q: ~149460000
**1**	6690000	6710000	**3**	q: ~ 81065000
**3**	3290000	3370000	**1**	q: ~76 815 000
**2**	74715000*		**Z**	56065000
**4**	45000	110000	**24**	pter: 0 - 20000
**4**	19905000	19910000	**Z**	p: ~ 8450000
**5**	21170000*		**20**	~ 2075000
**13**	15325000*		**27**	~ 65000
**Z**	38685000*		**14**	~14315000
**Z**	81855000	82005000	**3**	p: ~2200000
**Z**	81855000	82005000	**24**	qter: ~6270000

* - small ambiguous translocation of ~5 kb regions.

Additional Table 3. Intrachromosomal translocations identified in the HD3 cell line based on the analysis of Hi-C heatmaps.

**Chromosome #**	**start, ± 5000 bp**	**end, ± 5000 bp**	**SV type, comment**
**1**	180 580 000	194 260 000	duplication, associated with t(1;2)
**1**	194 265 000	196 140 000	partial deletion
**2**	25 780 000	25 875 000	deletion
**2**	54 325 000	61 685 000	partial deletion
**3**	0	2 195 000	partial deletion, associated with t(3;24)
**4**	16 840 000	18 830 000	partial deletion, associated with t(4;5), t(4;6), t(4;7), t(4;19)
**5**	605 000	795 000	partial deletion
**7**	0	4 105 000	partial deletion, associated with t(7;33)
**7**	9255000-9255000	10745000	small translocation
**9**	6070000	6990000	duplication
**11**	19 170 000	20 175 000	amplification
**17**	4 780 000	5 025 000	partial deletion
**21**	0	325 000	duplication
**22**	45 000	760 000	partial deletion
**24**	0-6275000*		duplication/amplification
**27**	0-5035000		duplication/amplification
**27**	5 040 000	5 645 000	partial deletion
**W**	0-5155000*		duplication

* - copy number variation involving the whole chromosome.
